# Dysfunction of the antisiphon device, SiphonGuard, due to rupture of the shell

**DOI:** 10.1186/1743-8454-7-S1-S21

**Published:** 2010-12-15

**Authors:** Kai Arnell, Pelle Nilsson

**Affiliations:** 1 Department of Paediatric Surgery1, University Hospital, Uppsala, Sweden

## Background

Codman Hakim programmable valves are used in treating hydrocephalic children in many countries. To prevent or treat over drainage with slit ventricle syndrome an antisiphon device (ASD) can be added. The ASD can be included in the valve or separate as the SiphonGuard.

## Materials and methods

A hydrocephalic boy shunted with a programmable Codman Hakim valve and a SiphonGuard has had two episodes of shunt dysfunction. The reasons were rupture of the ASD shell, which was difficult to detect. The medical records and x-rays were investigated.

## Results

At the first episode the boy was irritated and developed a minor swelling around the valve and the ASD. No disconnection of the shunt system was found at the plain x-ray. The symptoms accelerated and the boy was sent to a neurosurgical department for further investigation. A new plain x-ray verified the defect ASD even notable at the first investigation. At the second episode the symptoms were diffuse with variations in severity. Investigations showed no disconnection and normal CT scan. At the subsequent follow up, the symptoms became more pronounced especially in the mornings and a mild papilloedema was found. The valve was easy to press and filled quickly, why another follow up after adjusting to a lower opening pressure was recommended. At this follow up papilloedema with a little hemorrhage in the optic disc and a slight change in sensation at testing the valve was noticed. Exploration was decided and a rupture of the ASD shell was found. When aware of it, it was detectable on previous plain x-ray even if the picture was not perfect.

## Conclusions

In children with symptoms of shunt dysfunction it is important to have a plain X-ray of the whole shunt system, appropriate projections and be aware of how it should look, to be able to detect a defect SiphonGuard.

